# Editorial: Biomaterials with the regulation of reactive oxygen/nitrogen species for biomedical applications

**DOI:** 10.3389/fbioe.2022.1083727

**Published:** 2022-11-25

**Authors:** Qihui Zhou, Brandon W. Peterson, Yong Liu, Huihua Yuan

**Affiliations:** ^1^ Department of Stomatology, Institute for Translational Medicine, The Affiliated Hospital of Qingdao University, Qingdao University, Qingdao, China; ^2^ School of Rehabilitation Sciences and Engineering, University of Health and Rehabilitation Sciences, Qingdao, China; ^3^ Department of Biomedical Engineering, University of Medical Center Groningen, Groningen, Netherlands; ^4^ Wenzhou Institute, University of Chinese Academy of Sciences, Wenzhou, China; ^5^ School of Life Sciences, Nantong University, Nantong, China

**Keywords:** reactive oxygen species, reactive nitrogen species, biomaterials, anti-bacteria, anticancer, tissue repair and regeneration

In recent years, reactive oxygen/nitrogen species (ROS/RNS), as “two-faced” products, have garnered increasing interest in biomedical research (Patel et al., 1999; Pizzimenti et al., 2010; Sies and Jones, 2020). As is well-known, ROS/RNS can result in oxidative/nitrosative stress or the imbalance of redox homeostasis, damaging cellular macromolecules (e.g., lipids, protein, and DNA) and leading to the development of diseases (Patel et al., 1999; Checa and Aran, 2020). On the other hand, locally enhanced ROS/RNS can be used in the management of bacteria/virus infection and cancer (Dharmaraja, 2017; Yu et al., 2020; Tang et al., 2022). The precise regulation of ROS/RNS in living organisms and their surrounding microenvironment plays a crucial role in the treatment of diseases as well as in tissue repair and regeneration. The rational design and manufacture of advanced biomaterials has led them to emerge as a new ROS/RNS modulators, with significant potential in biomedical applications. In this Research Topic, 20 articles were published, focusing on the principles and applications of biomaterials for the regulation of ROS/RNS in the biomedical field.

In one of several reviews in this Research Topic, Shafiq et al. outlined the underlying mechanism of ROS formation in the cell/tissue microenvironment, and presented ROS-responsive biomaterials for tissue repair and regeneration applications. The authors highlighted the design and processing of ROS-scavenging functional biomaterials and their roles in both mediating oxidative stress and the damaged tissue/organ microenvironment for accelerating tissue repair.Ren et al.HYPERLINK Ren et al. reviewed the role of ROS in the bone microenvironment and outlined ROS-responsive biomaterials for the management of bone-related diseases. The authors proposed that bone-targeting biomaterials with ROS-responsive features and their accurate, controlled release should be particular areas of focus. Qi et al. HYPERLINK Qi et al. and Li et al. summarized the regulation properties of extracellular vesicles (EVs) and PEGylated catalases for ROS and oxidative stress, directly and indirectly. EVs containing antioxidant substances or oxidants can be delivered to receptor cells to accelerate or slow down oxidative stress. Meanwhile, regulators of oxidative stress-related signaling pathways can be mediated by EVs for delivery to receptor cells, allowing indirect regulation of oxidative stress. PEGylated catalases effectively modulated cytokine production by activating leukocytes, and inhibited elevated TNF-α and IL-6 levels in mice, inducing sepsis and considerably improving mouse survival rates. Furthermore, Sołtan et al. presented the main pathways of ROS/RNS regulation in tissue microenvironments and summarized recent advances in ROS/RNS-controlled biomaterials used in medicine. Jiang et al. summarized recent advances in nanomaterials with the capacity for ROS “turn-on” responses. Future work on the development of ROS “turn-on” detection nanomaterials should concentrate on enhancing the sensing capacity, detection limit, cytocompatibility, living cell imaging, and even and the ability to sample *in vivo*.

In an original research article in this Research Topic, regarding the regulation of biomaterial-mediated ROS for tissue repair and regeneration, Yang et al. designed porous Se@SiO_2_ nanoparticles which were applied to promote wound healing by mediating the ROS-PI3K/Akt pathway to suppress the generation of ROS, modulating the behaviors of fibroblasts and reducing scar formation. Lin et al. developed a skeletal muscle-derived adhesive hydrogel containing ZnONPs (ZnONPs-Gel), which exhibited excellent biocompatibility, significantly inhibited ROS formation, and suppressed cell apoptosis, thereby accelerating the function recovery in spinal cord injury. In addition to the ROS-mediated inorganic nanoparticles used, Wang et al. reported the fabrication of biomimetic hyaluronic acid methacryloyl/gelatin methacryloyl/extracellular cartilage matrix hydrogels for the repair of damaged, perforated tympanic membrane. The introduction of hyaluronic acid methacryloyl generated a high water content and elasticity and it scavenged ROS. The addition of an extracellular cartilage matrix promoted the chondrogenic differentiation of stem cells. A typical marine polysaccharide fucoidan exhibiting an antioxidant function was reported to possess the ability to remove ROS. Fucoidan-based nanofibers, as a bioactive scaffold, were developed by Chen et al.

In contrast, ROS-inducing biomaterials have been commonly used in antimicrobial and cancer applications. For antimicrobial research, Huang et al. developed a light-responsive silk-based hemostatic film containing a photodynamic agent (Chlorin e6) for effective photodynamic antibacterial therapy *via* forming ROS upon near-infrared (NIR) irradiation, which offers great promise in treating infected wounds and related diseases. Wang et al. reviewed recent advances in nanomaterials for COVID-19 therapy through generating ROS/RNS. For anticancer research, Cheng et al. summarized recent progress in nanosonosensitizers, with ultrasound-induced ROS formation for cancer sonodynamic immunotherapy (CSI). The challenges and outlook for clinical use of nanosonosensitizer-based CSI were further discussed. Wang et al. reviewed the “two-faced” effects of ROS on anticancer immunity, including macrophage polarization, immune checkpoint blocking (ICB) therapy, T cell activation and expansion, as well as the induction of immunogenic cell death. Designing smart biomaterials to mediate dynamic changes in ROS/RNS for specific biomedical purposes is critical for biomedical applications.

In summary, the appropriate regulation of ROS/RNS in organisms and their surrounding microenvironment plays a critical role in treating infection/cancer-related diseases, as well as tissue repair and regeneration. In future, the rational design and preparation of ROS-responsive smart biomaterials could offer enormous potential in advanced biomedical applications.
